# Abstinence duration and psychopathology among addiction outpatients during 18 months of COVID-19

**DOI:** 10.3389/fpsyt.2024.1339730

**Published:** 2024-02-08

**Authors:** Constanza Daigre, Raul Felipe Palma-Álvarez, Marta Sorribes-Puertas, German Ortega-Hernández, Marta Perea-Ortueta, Elena Ros-Cucurull, Lidia Segura, Joan Colom, Maria Dolores Braquehais, Josep Antoni Ramos-Quiroga, Lara Grau-López

**Affiliations:** ^1^ Department of Psychiatry, Hospital Universitari Vall d’Hebron, Barcelona, Spain; ^2^ Department of Psychiatry and Forensic Medicine, Universitat Autònoma de Barcelona, Bellaterra, Spain; ^3^ Group of Psychiatry, Mental Health and Addiction, Vall d’Hebron Institut de Recerca (VHIR), Barcelona, Spain; ^4^ Biomedical Network Research Center on Mental Health (CIBERSAM), Barcelona, Spain; ^5^ Subdirecció general de Drogodependències, Agència de Salut Pública de Catalunya, Barcelona, Spain; ^6^ Galatea Care Programme for Sick Health Professionals, Galatea Clinic, Barcelona, Spain

**Keywords:** COVID-19, substance use disorder, abstinence, outcome, longitudinal study, mental disorder

## Abstract

**Background:**

The COVID-19 pandemic has impacted the mental health of patients with substance use disorder (SUD). However, few longitudinal studies have been done which examine associations between the pandemic, SUD patients’ mental health and their drug use.

**Objectives:**

This study aimed to examine duration of abstinence according to psychiatric status among SUD outpatients followed-up for 18 months from the pandemic related lockdown.

**Methods:**

A follow-up study of 316 SUD outpatients was undertaken. Sociodemographic features, and clinical and consumption related variables were recorded. Pre, during and post lockdown information was evaluated. Abstinence/substance use was monitored at the patient’s scheduled follow-up appointments, and psychiatric disorders and psychological variables were revaluated at 18 months.

**Results:**

Survival analyses were used to compare the duration of abstinence (in months) from the beginning of the lockdown. It was observed that 70% of patients consumed the main substance for which they were being treated at some point during the follow-up. Men, younger patients, those with more symptoms of anxiety and personality disorders, and patients who experienced increased craving during follow-up, showed shorter duration of abstinence. While patients who had previously maintained at least one year of abstinence, achieved better results.

**Conclusions:**

During the first year and a half of the pandemic, SUD outpatients presented alterations in mental health, such us anxiety, depression and maladaptive personality traits and a high rate of relapse. For this reason, despite the health and social crisis and their restrictive measures, a comprehensive treatment should be ensured.

## 1 Introduction

The COVID-19 pandemic has been a major health challenge and forced countries to introduce severe restrictive measures (1). In Spain, strict home confinement measures were implemented for almost three months beginning in March 2020. Over time, the measures were gradually relaxed or eliminated, including lockdown, social distancing, wearing masks in specific locations, and changes to working conditions. This situation has had a significant impact on the mental health of the global population, with depression and anxiety being the most commonly reported symptoms (2–6). A study involving a large sample of the general Catalan population reported a threefold increase in the prevalence of depressive disorders (23%) and anxiety disorders (26%) compared to pre-confinement levels (4). Studies describing the impact on mental health symptoms at the onset of the pandemic observed rates reaching 75% to 80% of the population (2, 4). Patients with substance use disorder (SUD) are particularly vulnerable to the psychological effects of the social stressors caused by the pandemic (7, 8). It has been observed that patients with SUD exhibited a varied progression during confinement, 25.2% maintained their consumption pattern, 36.9% worsened, and 37.9% showed improvement in their consumption status. Another longitudinal study conducted during the pandemic reported that patients treated for SUD exhibited significantly higher psychological distress than a reference group without SUD, as assessed by a Symptom Checklist questionnaire (8).

Additionally, SUD patients often suffer comorbid mental disorders such as mood or personality disorders. Despite the heterogeneity of psychiatric comorbidity among SUD patients, anxious and depressive symptoms/disorders and maladaptive personality traits stand out (9, 10). Several studies report mixed results on how psychiatric comorbidity impacts the course of SUD. Some researchers describe a worse prognosis, more likely to relapse and drop-out of treatment (11–13). In this context, a study conducted by the National Institute on Drug Abuse (NIDA) found that 25% of patients undergoing SUD treatment had a major depressive disorder. Furthermore, this disorder was linked to increased substance use after one year of treatment (9). Conversely, others studies indicate no significant differences between patients with comorbid mental health disorders and those without (14–16). For example, regarding current comorbid psychiatric diagnoses and the completion of a substance-free program in community therapy, no significant differences were identified (14).

Cross-sectional studies with SUD patients have reported consistently high levels of psychological distress during the first months of the pandemic (15, 16). Among patients with alcohol use disorder, 50.3% of the sample reported a deterioration in depression and anxiety symptoms during lockdown (15). Furthermore, it has been estimated that 40.4% of patients undergoing treatment for various substances experienced a psychopathologic worsening global (17). Furthermore, despite lockdown and restrictions, a significant number of SUD patients maintained or worsened their consumption pattern (7, 18). There are few longitudinal studies on the SUD population and the course of SUD since the COVID-19 outbreak. One five-month follow-up study which compared psychological distress in SUD patients and the general population reported that loneliness predicted psychological distress in SUD patients (8). Similarly, another study conducted on the general population, reported that during the first year of the pandemic alcohol consumption increased among subjects who reported more psychological distress (19). Adding on, craving has been identified as a mediator between psychological problems and increased alcohol use in patients with alcohol use disorder (AUD) during the pandemic (20). However, one study among AUD patients did not find an association between lockdown measures and alcohol consumption (21). Another study found that patients whose mental health had worsened during the pandemic presented a stronger relationship between craving at baseline and substance use during the follow-up (22). Other substances have been less studied than alcohol. Regarding opioid use, one study described increased use among patients with opioid use disorder during the pandemic (23). Increased substance use may be partially explained by barriers to access addiction treatment as identified in prospective studies in SUD patients (24). In SUD patients, relapses are frequent and related to several factors, including biological, social and psychiatric factors (10, 16).

According to the above, to date there is consensus in the literature about the impact of the pandemic on the mental health of SUD patients. Nevertheless, most studies are cross-sectional and longitudinal data are scarce. Furthermore, prospective studies with SUD patients include small samples, focus specifically on alcohol, have a short follow-up time of only a few months, and do not include psychiatric comorbidity in the analysis and the evolution of anxiety and depression symptoms during the pandemic has not been studied.

Considering the above, it was hypothesized that the duration of abstinence after lockdown would be longer in patients with better psychiatric status and favorable previous treatment outcomes related to substance use. Hence, this study aims to compare the duration of abstinence in SUD outpatients, followed-up for 18 months from the outbreak of the pandemic, according to the psychiatric status. Additionally, it aims to identify substance-related and psychiatric state variables that independently associate with the duration of abstinence among treated patients.

## 2 Materials and methods

### 2.1 Study design and patients

An 18-month follow-up descriptive and analytical study was conducted between 03/15/2020 and 09/15/2021 on SUD patients who received outpatient treatment at the Addiction and Dual Diagnosis Unit of Vall d’Hebron Hospital, Barcelona, Spain. The patients participating in the study were undergoing treatment at the onset of the pandemic. Pre, during and post lockdown information was evaluated. Inclusion criteria were patients with a diagnosis of SUD, aged older than 18 years. Patients whose low Spanish proficiency interfered with their ability to understand the study proposal were excluded. The project was approved by the Ethics Committee of Vall d’Hebron Hospital (PR-(AG)386-2020). Patients did not receive any financial compensation and written informed consent was obtained from all participants. In the case of phone call visits during lockdown, consent was procured orally and ratified in writing during the following in-person visit.

### 2.2 Procedure and characteristics of the treatment center

Interviews were conducted by the trained psychiatrists and psychologists responsible for each participant. During the lockdown in Barcelona an *ad hoc* interview prepared for the study was used which recorded sociodemographic features, and clinical and consumption related variables. The inclusion and assessment interviews were undertaken either during in-person visits or telematically. Substance use was then monitored in the programmed follow-up appointments by the patient’s main therapist. At 18 months, psychiatric disorders and psychological variables were revaluated.

The center primarily serves to residents of the northern districts of Barcelona. The therapy team comprises psychiatrists, psychologists, nurses, and addiction-specialized social workers. The outpatient clinic provides treatment involving individual psychotherapy and psychopharmacological interventions addressing both substance use and psychiatric comorbidities. At a therapeutic level, a personalized intervention is conducted, including common topics present in addiction treatment, such as motivation for change, relapse prevention, emotional regulation, and social context. The psychotherapeutic sessions and psychiatric appointments are scheduled at least once a month and the frequency varies according to each patient’s needs (between weekly and monthly).

### 2.3 Instruments and variable

#### 2.3.1 Sociodemographic and clinical features

An *ad hoc* interview was used to record sociodemographic and clinical data at the time of enrolment. Sociodemographic features recorded were gender, age, nationality, educational level, civil status, housing, employment status, and criminal record. Information regarding SUD included history of SUD, polysubstance use (understood as three or more SUDs), use of injecting, previous SUD treatments, and length of abstinence prior to lockdown. Previous co-occurring psychiatric disorders were assessed by a trained psychiatrist or clinical psychologist and established by clinical judgment, following the DSM-5 criteria (25).

#### 2.3.2 Substance use status and psychopathological variables during lockdown and at the follow-up

Substance use pattern was assessed by the same *ad hoc* questionnaire, during lockdown. This questionnaire included evaluation of changes in substance use during lockdown, psychiatric status, and COVID-19 related variables. Compliance with the rules during lockdown and the subsequent relaxation of the rules was also evaluated. Psychopathological variables were evaluated during lockdown and revaluated at 18-month follow-up. Feelings of loneliness reported by the patients were recorded. Mental disorders other than SUD were assessed by clinical judgment, following the DSM-5 criteria (25). Mental disorders were grouped by psychotic, depressive, anxiety, and personality disorders. Psychiatric emergency room visits and psychiatric hospitalization were also recorded. General psychiatric status and psychological variables were assessed using the following instruments.

- The Clinical Anxiety Scale (CAS) is a hetero-applied scale which measures anxiety symptoms using 7 items scored on a 5-point Likert scale (0–4). Five or more points indicate the presence of anxiety symptoms mild, moderate, or severe. It has adequate validity, reliability, and sensitivity to change (26).
- The Brief Psychiatric Rating Scale which assesses 18 symptom domains through clinical judgment and questioning, using a Likert scale from 0 (not present) to 6 (extremely severe). The evaluated domains are: somatic concern, anxiety, emotional withdrawal, conceptual disorganization, guilt feelings, tension, mannerisms and posturing, grandiosity, depressive mood, hostility, suspiciousness, hallucinatory behaviour, motor retardation, uncooperativeness, unusual thought content, blunted affect, excitement, and disorientation. Due to its clinical significance, the domain of depressive mood was described separately, identifying the presence of positive depressive symptoms with a score of 3 or more (27).
- Loneliness: The Three-Item Loneliness Scale is an interviewer-administered questionnaire developed from the Revised UCLA Loneliness Scale. Each question is rated on a 3-point Likert scale (1–3). All items are summed to give a total score. The scale provides a succinct method to collect information about social isolation. During lockdown feelings of loneliness were evaluated by a dichotomous question (yes/no) included in the *ad hoc* interview (28).
- Overall psychiatric severity was evaluated through the Clinical Global Impression-Severity scale (CGI-S) (29), which uses a Likert scale (0-7 points). Scores 1-4 represent normal to moderately ill and scores 5-7 represent markedly to extremely ill (30).

#### 2.3.3 Abstinence during the follow-up

The principal substance of use identified by the patient’s main therapist was evaluated. Abstinence was assessed once a month at the standard follow-up appointments. The duration of abstinence was measured in months until the first use of the main substance. Using once or more was considered a relapse (i.e. the end of abstinence).

### 2.4 Statistical analysis

Descriptive statistics were calculated for the main variables. Survival analyses were used to compare the duration of abstinence (in months) from the beginning of the lockdown to 18-month follow-up. Kaplan–Meier estimates were conducted to obtain bivariate comparisons, for which the log-rank test was used. To reduce false-positive results, the Bonferroni correction for multiple tests was performed according to the number of tests in each group of bivariate analysis. Two multivariate analysis models were executed using Cox regression analyses, one for substance-related variables and another for psychiatric status. Only variables that retained statistical significance after the Bonferroni correction were included and both models included age and gender variables. Assessment of anxiety, general psychiatric status and clinical global impression, during lockdown and during follow-up, were compared. Because these variables had a nonparametric distribution tested by the Kolmogorov–Smirnov test, the Wilcoxon test was used for comparing the two paired quantitative variables and the NcNemar test for frequencies. All statistical hypotheses were two-tailed. SPSS, version 20 for Windows, was used for all analyses.

## 3 Results

**Figure 1:**
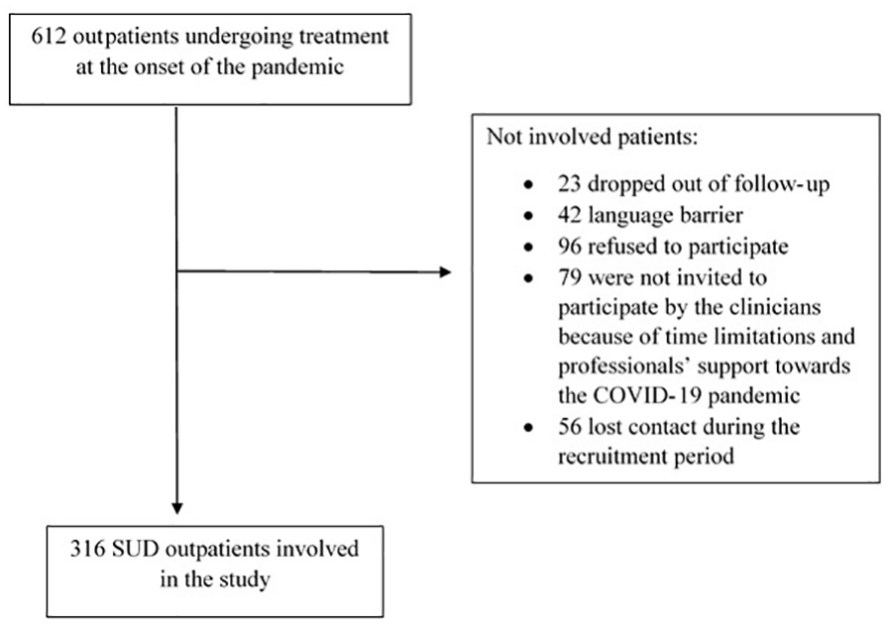
Study flowchart.

As **Figure 1** shows, from a potential sample of 612 patients, 316 SUD outpatients participated were included in the final follow-up at 18 months (71.3% males; mean age 48.4 ± 11.7). The mean duration of abstinence for the total sample was 7.9 ± 7.6 months. The average adherence to treatment was 14.1 ± 6.0 months. **Table 1** shows sociodemographic features and substance related variables. The most frequent SUD was AUD, followed by opiate and cocaine use disorders. At the start of the pandemic, 36.7% of the patients had been abstinent from substance use for more than a year and 71.6% had been diagnosed with another mental disorder in addition to SUD (See **Table 1**).

**Table 1:** Sociodemographic characteristics and substance related variables.

Sociodemographic characteristics	
Gender (male)	233 (73.7%)
Mean age (years)	48.6 ± 11.7
Spanish nationality	288 (88.1%)
Secondary or higher education	147 (45%)
Civil status (married or partner)	130 (39.8%)
Living with their family	230 (70.3%)
Employed	81 (24.8%)
Criminal records	106 (32.4%)

Main Substance Use Disorders (SUD)	
Opiate use disorder	90 (28.1%)
Cocaine use disorder	62 (19.4%)
Alcohol use disorder	133 (41.6%)
Benzodiazepine use disorder	17 (5.3%)
Cannabis use disorder	18 (5.6%)
Tobacco use disorder	285 (87.2%)
Polysubstance use disorders	145 (44.3%)
Injecting drug use	70 (21.4%)
Previous treatment for SUD	270 (82.6%)
Abstinence for more than a year	120 (36.7%)
Dual diagnosis	234 (71.6%)

**Table 2** shows SUD related variables, data related to adaptation to the pandemic and psychiatric status during follow-up. It was identified that 14.2% of patients maintained active consumption, 31.2% worsened their consumption pattern and in 54.6% their SUD improved or maintained abstinence. Opiates and cocaine were the substances for which the most increases in use were observed. 30% of patients reported increased craving.

**Table 2:** Substance related variables, adaptation to pandemic restrictions and psychiatric status during follow-up.

Substance Use Disorders variables	
Consumption pattern evolution during follow-up	
Active consumption maintained	45 (14.2%)
Worsened consumption pattern	98 (31.2%)
Improved consumption or abstinence	172 (54.6%)
Substance use increases	
Opiate use increases in OUD	25 (20.5%)
Cocaine use increases in CUD	48 (15.3%)
Alcohol use increases in AUD	65 (20.7%)
Benzodiazepine use increases in BUD	32 (10.2%)
Cannabis use increases in CNUD	22 (7%)
Tobacco use increases in TUD	40 (12.7%)
Increased craving during follow-up	96 (30.7%)
Adaptation to pandemic restrictions	
Broke the lockdown norm or restrictions	63 (24.2%)
Difficulty in adapting to relaxed restrictions	16 (17.7%)
Worsened family relationship	65 (22.1%)
Serious medical illness	70 (24.4%)
Psychiatric Status	
Psychiatric disorder during follow-up	190 (63.8%)
Psychotic disorder	53 (17.8%)
Depressive disorder	73 (24.5%)
Anxiety disorder	56 (18.8%)
Personality disorder	67 (25%)
Psychiatric emergencies	31 (10.5%)
Psychiatric hospitalization	9 (3.1%)
UCLA Loneliness Scale	80 (33.3%)
Clinical Anxiety Scale (CAS ≥5)	143 (49.7%)
Brief Psychiatric Rating Scale (above average 27)	91 (31.6%)
Depressive symptoms in BPRS follow-up (≥3)	125 (43.4%)
Clinical Global Impression-Severity scale (markedly to extremely ill)	37 (11%)

OUD, Opiate Use Disorder; CUD, Cocaine Use Disorder; AUD, Alcohol Use Disorder; BUD, Benzodiazepine Use Disorder; CNUD, Cannabis Use Disorder; TUD, Tobacco Use Disorder; BPRS, Brief Psychiatric Rating Scale.

**Table 3** shows the survival analysis results of the duration of abstinence during follow-up according to the sociodemographic and substance related variables. It was observed that younger and foreign patients achieved shorter duration of abstinence. Alcohol was the substance associated with longer duration of abstinence and cannabis to shorter duration. Patients who experienced higher craving levels and had not been abstinent for more than a year at the start of the lockdown achieved shorter duration of abstinence.

**Table 3:** Duration of abstinence in months from lockdown according to sociodemographic features and substance related variables.

	Mean (months ± TE)	Mean (months ± TE)	X^2^	p
Sociodemographic features				
Gender (male vs female)	7.2 ± 0.5	9.5 ± 0.8	5.1	0.024
Age (Under/above average 48 yrs)	6.2 ± 0.6	9.1 ± 0.6	12	<0.001*
Nationality (Spanish/other)	6.9 ± 0.3	3.6 ± 0.7	14.8	<0.001*
Secondary or higher vs primary education	7.5 ± 0.6	8.1 ± 0.6	0.8	0.378
Marital status (partner vs no partner)	8.4 ± 0.7	7.4 ± 0.6	0.9	0.338
Living with their family vs alone	8.4 ± 0.5	6.4 ± 0.7	4.2	0.042
Employed vs unemployed	7.1 ± 0.8	8.1 ± 0.5	1.6	0.199
Criminal record vs no	6.4 ± 0.7	8.5 ± 0.5	4.7	0.030
Substance related variables				
**Main Substance Use Disorders (SUD)**			15.4	0.004*
Opiate use disorder	7.8 ± 0.8			
Cocaine use disorder	6.4 ± 0.9			
Alcohol use disorder	9.2 ± 0.7			
Benzodiazepine use disorder	7.1 ± 1.9			
Cannabis use disorder	3.2 ± 1.2			
Tobacco use disorder vs no	7.6 ± 0.5	9.6 ± 1.1	1.8	0.178
Polysubstance use disorders vs no	6.6 ± 0.6	8.8 ± 0.6	4.9	0.027
Injecting drug use vs no	6.4 ± 0.9	8.2 ± 0.5	2.3	0.130
Increased craving (yes vs no) during follow-up	3.9 ± 0.5	9.7 ± 0.5	51.7	<0.001*
Previous treatment for SUD vs no	8.2 ± 0.5	6.1 ± 0.9	5.8	0.016
Abstinence for more than a year vs no	11.8 ± 0.6	5.6 ± 0.4	45.6	<0.001*

*Significant after Bonferroni correction, X^2^Chi square log rank test for survival analysis.

According to log rank test for survival analysis.

**Table 4** shows the survival analysis results of the duration of abstinence during follow-up according to the adaptation to pandemic restrictions and psychiatric status during follow-up. Patients who reported having breached the lockdown or subsequent rules and who worsened their family relationships-maintained abstinence for significantly less time. Regarding psychiatric status, having been diagnosed with any psychiatric disorder or presenting with any personality disorder during follow-up was associated with shorter abstinence duration. Evaluation of symptoms that may be subthreshold or that do not constitute a psychiatric disorder showed that patients with more feelings of loneliness, more anxiety symptoms measured by CAS (a score of 5 or more indicates mild, moderate, or severe anxiety, more depressive symptoms measured by BPRS (a score of 3 or more indicates mild, moderate, or severe depressive symptoms) and greater global severity had shorter duration of abstinence.

**Table 4:** Duration of abstinence in months from lockdown according to adaptation to pandemic restrictions and psychiatric status during follow-up.

	Mean (months ± TE)	Mean (months ± TE)	X^2^	p
Adaptation to pandemic restrictions				
Breach the lockdown norm or restrictions vs no	4.3 ± 0.8	10.1 ± 0.5	33.8	<0.001*
Difficulty adapting to relaxing restrictions vs no	8.7 ± 1.1	8.7 ± 0.5	0	0.958
Worsened family relationship vs no	6.1 ± 0.8	8.9 ± 0.5	9.1	0.003*
Psychiatric Status				
Previous Dual diagnosis vs no	7.7 ± 0.5	8.1 ± 0.8	0.1	0.918
Any psychiatric disorder during follow-up vs no	7.3 ± 0.5	9.9 ± 0.7	8.7	0.003*
Psychotic disorder vs no	7.3 ± 1.1	8.4 ± 0.5	0.3	0.603
Depressive disorder vs no	7.8 ± 0.8	8.4 ± 0.5	2.032	0.154
Anxiety disorder vs no	8.3 ± 0.8	8.2 ± 0.5	0.1	0.705
Personality disorder vs no	5.5 ± 0.8	9.3 ± 0.5	12.4	<0.001*
Psychiatric hospitalization vs no	5.3 ± 2.4	8.4 ± 0.4	1.1	0.288
UCLA Scale (loneliness vs no)	6.7 ± 0.8	9.6 ± 0.6	8.2	0.004*
Clinical Anxiety Scale (Anxiety symptoms vs no)	10.2 ± 0.6	6.8 ± 0.6	14.4	<0.001*
Brief Psychiatric Rating Scale (above average 27)	6.9 ± 0.7	9.2 ± 0.5	7.5	0.006
Depressive symptoms in BPRS (yes vs no)	6.7 ± 0.6	9.8 ± 0.6	13.6	<0.001*
Clinical Global Impression-Severity scale (markedly to extremely ill vs less severity)	3.6 ± 1	8.46 ± 0.5	14.5	<0.001*

*Significant after Bonferroni correction. X^2^Chi square log rank test for survival analysis.

According to log rank test for survival analysis.

Two Cox regression models were conducted using the variables that retained statistical significance after the Bonferroni correction and considering the duration of abstinence in months as the dependent variable (**Table 5**). Therefore, despite showing significant differences at the bivariate level, the variables living with their family, criminal record, polysubstance use, and previous treatment were not included in the model. The model related to substance-related variables showed that the age, increased craving, and having remained abstinent for more than a year were independently associated with duration of abstinence during follow-up (X2 = 69.3;p=<0.0001). The other model showed that the male gender, comorbidity with personality disorders and anxiety symptoms (CAS) were independently associated with duration of abstinence during follow-up (X2 = 34.8;p=<0.0001).

**Table 5:** Results of Cox regression regarding duration of abstinence.

	Wald	p	Hazard ratio	95.0% CI	
				Lower	Upper
Model 1: substance-related variables					
Gender (male)	1.918	0.166	1.248	0.912	1.708
Age (Under average 48 yrs)	3.906	0.048	1.316	1.002	1.727
Main Substance Use Disorders	1.312	0.252	1.074	0.950	1.215
Increased craving during follow-up	21.202	<0.001	0.510	0.383	0.679
Abstinence for more than a year at the pandemic beginning	18.598	<0.001	2.080	1.491	2.901
Model 2: Psychiatric status variables during follow-up					
Gender (male)	6.308	0.012	1.660	1.118	2.465
Age (Under average 48 yrs)	2.118	0.146	1.291	0.915	1.823
Any psychiatric disorder during follow-up	0.005	0.942	.984	0.639	1.515
Personality disorder	7.270	0.007	0.591	0.403	0.866
Loneliness in UCLA Scale	2.012	0.156	0.761	0.522	1.110
Anxiety symptoms in CAS	5.043	0.025	0.645	0.439	0.946
Depressive symptoms in BPRS	0.044	0.834	1.041	0.712	1.524

CAS, Clinical Anxiety Scale; BPRS, Brief Psychiatric Rating Scale; Degrees of freedom=1.

Baseline and follow-up measures of anxiety, general psychiatric status, loneliness, and overall psychiatric severity were compared. At 18 months a decrease in anxiety symptoms (CAS) and an increase in depressive symptoms (BPRS) were observed. These averages correspond to a decrease from 52.5% to 49.7% of patients reporting anxiety symptoms and an increase from 32.6% to 43.4% of those reporting depression symptoms. No significant changes were observed regarding the percentage of patients who reported feelings of loneliness using the UCLA Loneliness Scale. The CGI-S showed significantly greater severity at the reassessment (See **Table 6**).

**Table 6:** Comparison between the symptoms measured during the lockdown and at 18 month follow-up.

	Basal	Follow-up		
	Mean ± SD	Mean ± SD	Z	p
Clinical Anxiety Scale (CAS)	6,3 ± 5.7	5,5 ± 5.3	2.2	0.029
Brief Psychiatric Rating Scale (BPRS)	25,6 ± 7.8	26,7 ± 8.9	1.1	0.252
Depressive symptoms in BPRS	2 ± 1.4	2,4 ± 1.5	3.4	0.001
Clinical Global Impression-Severity scale	2,9 ± 1.5	3,4 ± 1.6	5	<0.001
Loneliness in UCLA Loneliness Scale	36.2%	33.6%		0.053

## 4 Discussion

The study results showed that several sociodemographic and clinical factors were associated with duration of abstinence in SUD outpatients during the pandemic. This study showed that men, younger patients, patients with more anxiety and personality disorders, and patients who had increased craving during follow-up, had shorter duration of abstinence. Patients who had previously maintained at least one year of abstinence, achieved better results.

In line with previous studies of SUD outpatients, relapses were found to be frequent throughout follow-up. In the current study, 70% of the patients consumed the main substance at some point during the 18 months of follow-up. Although there is no pre-pandemic control group and the design of this study differs from previous ones, these results seem to be somewhat lower than in previous studies in the same setting (50% relapsed within 6 months; 80% within 1 year) (10, 31). Several factors may be related to lower consumption during this stage of the health crisis, such as restricted access to drugs during lockdown or subsequent measures regarding travel and social distancing with limited social gatherings. Nonetheless, it highlights the importance of treatment centers having prepared action protocols to maintain psychiatric and psychological follow-up. For example, this may include telemedicine interventions to continue working on motivation for change and improving the quality of life for addicted patients, as they serve as indicators of progress (32–34).

Regarding sociodemographic results, male patients presented earlier relapses and the multivariate analysis about psychiatric status found an independent association with gender. In the general population, alcohol consumption has been identified as a coping strategy more prevalent in males during the pandemic (35). Furthermore, it has also been described that during the lockdown in Catalonia, male patients more frequently consumed cocaine and alcohol than females (17). There are likely multiple factors associated with the difference in treatment outcomes based on gender during the pandemic. However, it is possible to hypothesize that women may have more coping strategies to alleviate psychological distress, leading to less frequent substance use. The caregiving role often associated with women may act as a protective factor. Nevertheless, it is essential to maintain a gender perspective to provide treatment according to the specific needs of women, who tend to be more stigmatized and experience a lower quality of life in cases of dual pathology (34).

Younger age was also independently associated with shorter duration of abstinence; this finding is coherent with studies prior to and during the pandemic that report worse treatment results in younger patients (7, 17, 36). More impulsivity and more psychological distress during the pandemic could be related to younger age (37, 38). Being a foreigner was also associated with shorter abstinence duration. Although several factors may be related to this result, this group having worse social support during the pandemic could partially explain this finding (39, 40).

Concerning the main substance in the bivariate analysis, patients who started treatment for alcohol consumption-maintained abstinence for longer during follow-up. Conversely, cannabis as the main substance of treatment was associated with shorter abstinence. These results differ from the increase in alcohol consumption described in the general population during the pandemic and are more similar to the results of a longitudinal study among AUD patients that did not find an increase in alcohol consumption during the lockdown (21). The shorter duration of abstinence among those with cannabis as their main substance could be explained by the lower perception of risk described in these patients (41, 42). Furthermore, during the early stages of the pandemic an increase was seen in cannabis use among daily users, especially in those with greater previous severity, with greater feelings of loneliness, and as a coping strategy (7, 43, 44). It is important to mention that the main SUD does not maintain statistical significance in the regression model of substance-related variables. This suggests that the main SUD is not a determining factor associated with the evolution during follow-up. Instead, the increase in craving and maintaining abstinence for a year before the beginning of the pandemic are the significant variables after multivariate analysis.

Recovery from SUD is a long process that involves improvements in wellbeing. It has been identified that during the first five years of abstinence the most critical changes occur (45). Congruently, it was observed that patients who had maintained abstinence for more than one year prior to lockdown showed better outcomes in the multivariate analysis. Patients who had acquired more strategies and who were more motivated to achieve abstinence obtained better results during follow-up.

As in other studies, our logistic regression analysis results, confirmed the expected associations between the increase in craving during follow-up and earlier relapse (46). Craving has been identified as a mediating factor between psychological problems and increased alcohol use in AUD patients during the pandemic (20). The treatment of craving is essential for a comprehensive approach to addictions. This outcome emphasizes the importance of focusing on acceptance, identification, prevention, and coping with the desire for substance use during periods of social and health crises (47, 48).

Patients who reported having breached the lockdown or subsequent rules and whose family relationships worsened maintained abstinence for less time. Most of the COVID-19 related restrictions implemented during the follow-up were related to movement (e.g. home confinement during the day or at night) and the use of masks. It was expected that active users would have more difficulties in complying with these restrictions. In the same way, patients who relapsed earlier reported more family difficulties. The serious family problems caused by addictions are well known (49).

Patients with pre-existing mental disorders have been identified as an at-risk group for adverse outcomes both physical and emotional (50, 51). In our current study, patients with psychiatric comorbidity achieved shorter duration of abstinence. Studies prior to the pandemic have pointed to the relationship between psychiatric status and the course of addiction (10, 52–54). However, this relationship has been minimally studied during the pandemic. Regarding psychiatric status, personality disorders and anxiety stand out in the multivariate analysis. Patients with personality disorders relapsed earlier. Abstinence maintenance can be complicated maladaptive personality traits, such as high impulsivity, risk-taking behavior, and difficulties in adapting to norms and regulating emotions (55–57). On the other hand, anxiety in SUD patients was frequent during the pandemic. In pre-pandemic studies, anxiety was independently associated with shorter duration of abstinence. The emotional discomfort caused by anxiety symptoms during the pandemic could have precipitated consumption in SUD patients, in line with the theory of self-medication. It is also possible that patients with greater anxiety have fewer resources to manage craving and self-control (58, 59). Despite not maintaining statistical significance in the multivariate analysis, associations between duration of abstinence and depressive symptoms, feelings of loneliness and overall psychiatric severity were significant in bivariate analysis. Depressive symptoms, whether they are the primary disorder or consequences of substance use, are prevalent among SUD patients and have a great impact on the course of treatment (32, 60, 61). Furthermore, for many individuals, social distancing during the pandemic led to social isolation, increasing symptoms of depression or poor mental health (62). Perceived loneliness is of great relevance since has been identified as a main predictor of mental health, which can affect the course of addiction (63). Similarly, in vulnerable populations to the impact of COVID, the role of perceived loneliness has been highlighted as a determinant of emotional well-being (64). Among patients with dual diagnosis, specifically those with schizophrenia, social support as a coping strategy has been identified as a protective factor against relapses at the twelve-month follow-up (65). Likewise, patients that described more social isolation during COVID-19 lockdown had more difficulties to cope negative emotions (66). All this is consistent with the fact that patients with greater clinical severity relapsed earlier than patients identified as less severe. Therefore, a comprehensive approach, including the treatment of SUDs together with other psychiatric symptoms, such as maladaptive personality traits, anxiety, and depressive and loneliness feelings, is a challenge for therapeutic teams (33).

Regarding the comparison of the psychological scales applied at the beginning and at the 18 month follow-up, the results showed a decrease in anxiety, but an increase in depressive symptoms and a general worsening measured by CGI-S. In the case of anxiety and depressive symptoms in the SUD patients studied, their evolution is consistent with studies which described increased anxiety in 2020 and an improvement in 2021 or 2022 (67). Moreover, when the stressful situation is prolonged, an increase in depressive symptoms is expected (68, 69).

Some limitations should be considered. Firstly, relapse was self-reported and no biological test was systematically used. Albeit, there is a high agreement between self-reported substance use and urinalysis results (70). Also, the pandemic has changed the dynamics on urinalysis controls and how urinalysis is conceived in addiction treatment (71). Secondly, only two assessments were conducted during the 18-month follow-up of psychiatric symptoms, making it challenging to associate these symptoms with restrictions arising from the health crisis. Moreover, feelings of loneliness were evaluated with the UCLA scale only during the follow-up, while the baseline evaluation was conducted by clinical assessment. Therefore, the validity of these results should be interpreted with caution. However, abstinence maintenance was evaluated monthly during outpatient visits. A third limitation of the study is that gender differences were not systematically described, as the decision was made to focus the information on abstinence time to enhance comprehension of results. Finally, this study was carried out at a single center; the generalization of the results is limited. However, the sample is large and includes patients treated for consumption of all the usual substances consumed in Spain. When it comes to the strengths of the study, the long follow-up period, should be highlighted as well as inclusion of patients addicted to different substances, and the assessment of the concurrence with other disorders and psychiatric symptoms throughout the pandemic. In the field of addictions, where the objective is often on achieving and maintaining abstinence, longitudinal studies, such as the one presented, offer valuable insights for designing and implementing interventions in various contexts.

Finally, the results of the study indicate that the duration of abstinence during the first year and a half of the COVID-19 pandemic was shorter in patients with SUD who experienced an increase in craving, had a worse pre-pandemic course, were male, had personality disorders, and higher levels of anxiety. Additionally, anxiety symptoms showed a decrease, while depressive symptoms increased over the follow-up period. These findings suggest that special attention should be paid to those patients presenting specific risk factors identified in the study. These patients may benefit from personalized interventions and additional support during critical periods. This could involve therapeutic strategies addressing craving management, strengthening coping skills, and enhancing overall emotional well-being. Moreover, the increase in depressive symptoms throughout the follow-up underscores the importance of continuous monitoring of the mental health of these patients, even after initial periods of stability. Mental health professionals should remain vigilant to potential changes in symptoms and adjust interventions accordingly, ensuring a comprehensive and tailored treatment considering their prior history, sociodemographic differences, and clinical characteristics.

## Data availability statement

The raw data supporting the conclusions of this article will be made available by the authors, without undue reservation.

## Ethics statement

The studies involving humans were approved by The Ethics Committee of Vall d’Hebron Hospital approved the study (PR- (AG)386-2020). The studies were conducted in accordance with the local legislation and institutional requirements. The participants provided their written informed consent to participate in this study.

## Author contributions

CD: Conceptualization, Data curation, Formal analysis, Investigation, Methodology, Project administration, Supervision, Writing – original draft, Writing – review & editing, Visualization. RP-Á: Conceptualization, Investigation, Methodology, Project administration, Supervision, Writing – original draft, Writing – review & editing. MS-P: Conceptualization, Investigation, Project administration, Supervision, Writing – review & editing, Methodology, Writing – original draft. GO-H: Investigation, Writing – review & editing, Supervision, Writing – original draft. MP-O: Conceptualization, Investigation, Methodology, Supervision, Writing – original draft, Writing – review & editing. ER-C: Conceptualization, Investigation, Methodology, Supervision, Visualization, Writing – review & editing. LS: Formal analysis, Investigation, Writing – original draft, Writing – review & editing. JC: Conceptualization, Supervision, Writing – original draft, Writing – review & editing. MB: Formal analysis, Investigation, Writing – review & editing. JR-Q: Conceptualization, Investigation, Resources, Supervision, Validation, Visualization, Writing – original draft, Writing – review & editing. LG-L: Conceptualization, Investigation, Methodology, Supervision, Visualization, Writing – original draft, Writing – review & editing.
